# Supplementary material for: Use of a mouse model for the isolation of Borrelia puertoricensis from soft ticks
Source: PLoS One. 2025 Feb 18;20(2):e0318652. doi: 10.1371/journal.pone.0318652 (PMC11835249; doi:10.1371/journal.pone.0318652)
Supplement: Attachment — Submitted filename: Rebuttal_letter.pdf [file pone.0318652.s002.pdf]

January 9th, 2025

Rebbutal letter

Dear Dr. Latrofa

Thank you for your message and for taking over our manuscript. Before I do any more comments I would llike to tell you that this paper was originally edited by Dr. Wolfram Zueckert but the reviewed version couldn't be uploaded because the Editorial Manager had a glitch that couldn't be fixed and thus the PLOS ONE office asked me to send it as a new submission, when actually it wasn't. Anyhow, I believe you needed to know this as thgis explains a bit my insistence.
Here I answer your comments, those made by the Journal and those by the reviewer, all are greatly appreciated for their efforts in this process. Please note that the reponses are marked as such and are placed immediately below the corresponding comment/suggestion.
Please do not hesitate to contact me in case there is any questions regarding this submission. I also take this opportunity to thank you and the reviewer for your comments.

Best regards,

Antonio

---*---
PONE-D-24-47355
Use of a mouse model for the isolation of *Borrelia puertoricensis* from ticks collected in Merida, Yucatan, Mexico
PLOS ONE

Dear Dr. Ibarra,

Thank you for submitting your manuscript to PLOS ONE. After careful consideration, we feel that it has merit but does not fully meet PLOS ONE's publication criteria as it currently stands. Therefore, we invite you to submit a revised version of the manuscript that addresses the points raised during the review process.

Please submit your revised manuscript by Feb 18 2025 11:59PM. If you will need more time than this to complete your revisions, please reply to this message or contact the journal office at plosone@plos.org. When you're ready to submit your revision, log on to https://www.editorialmanager.com/pone/ and select the 'Submissions Needing Revision' folder to locate your manuscript file.

Please include the following items when submitting your revised manuscript:

A rebuttal letter that responds to each point raised by the academic editor and reviewer(s). You should upload this letter as a separate file labeled **'Response to Reviewers'**.
A marked-up copy of your manuscript that **highlights** changes made to the original version. You should upload this as a separate file labeled **'Revised Manuscript with Track Changes'.**
An unmarked version of your revised paper without tracked changes. You should upload this as a separate file labeled **'Manuscript'**.
If you would like to make changes to your financial disclosure, please include your updated statement in your cover letter. Guidelines for resubmitting your figure files are available below the reviewer comments at the end of this letter.

If applicable, we recommend that you deposit your laboratory protocols in protocols.io to enhance the reproducibility of your results. Protocols.io assigns your protocol its own identifier (DOI) so that it can be cited independently in the future. For instructions see: https://journals.plos.org/plosone/s/submission-guidelines#loc-laboratory-protocols. Additionally, PLOS ONE offers an option for publishing peer-reviewed Lab Protocol articles, which describe protocols hosted on protocols.io. Read more information on sharing protocols at https://plos.org/protocols?utm_medium=editorial-email&utm_source=authorletters&utm_campaign=protocols.

We look forward to receiving your revised manuscript.
Kind regards,

Maria Stefania Latrofa
Academic Editor
PLOS ONE

Journal Requirements:

When submitting your revision, we need you to address these additional requirements.

1. Please ensure that your manuscript meets PLOS ONE's style requirements, including those for file naming. The PLOS ONE style templates can be found at https://journals.plos.org/plosone/s/file?id=wjVg/PLOSOne_formatting_sample_main_body.pdf and https://journals.plos.org/plosone/s/file?id=ba62/PLOSOne_formatting_sample_title_authors_affiliations.pdf

RESPONSE: to the best of my knowledge I have followed the style requirements of this journal.

2. In your Methods section, please provide additional information regarding the permits you obtained for the work. Please ensure you have included the full name of the authority that

approved the field site access and, if no permits were required, a brief statement explaining why.

RESPONSE: Animal procedures were aproved by our ethics committee and this has been mentoioned in the document (lines 139-142). As for the collection of the ticks, no permit was required as ticks are not endangered species and the collection was done in a public park without disturbing any sections of this small park. This comment has been added to the manuscript in lines 138 to 142.

3. Thank you for stating the following financial disclosure:
This work was supported by funds to JAI from Secretaría de Investigación y Posgrado-IPN (20230850, 20240122, 20241496) and by funds provided to JEL from the National School of Tropical Medicine at Baylor College of Medicine.

Please state what role the funders took in the study. If the funders had no role, please state: ""The funders had no role in study design, data collection and analysis, decision to publish, or preparation of the manuscript.""
If this statement is not correct you must amend it as needed.
Please include this amended Role of Funder statement in your cover letter; we will change the online submission form on your behalf.

RESPONSE: We kindly request that the statement "The funders had no role in study design, data collection and analysis, decision to publish, or preparation of the manuscript." Thank you.

4. **Please include your full ethics statement in the 'Methods' section of your manuscript file.** In your statement, please include the full name of the IRB or ethics committee who approved or waived your study, as well as whether or not you obtained informed written or verbal consent. If consent was waived for your study, please include this information in your statement as well.

RESPONSE: As we used mice for our experiments the only ethics statement is already included in the manuscript. We have modified this section of the manuscript to mention that this is icnluded in the section where the animals are used (lines 138-142).

5. We note that your Data Availability Statement is currently as follows: All relevant data are within the manuscript and its Supporting Information files.

Please confirm at this time whether or not your submission contains all raw data required to replicate the results of your study. Authors must share the "minimal data set" for their submission. PLOS defines the minimal data set to consist of the data required to replicate all study findings reported in the article, as well as related metadata and methods (https://journals.plos.org/plosone/s/data-availability#loc-minimal-data-set-definition).

For example, authors should submit the following data:

- The values behind the means, standard deviations and other measures reported;
- The values used to build graphs;
- The points extracted from images for analysis.

Authors do not need to submit their entire data set if only a portion of the data was used in the reported study.

If your submission does not contain these data, please either upload them as Supporting Information files or deposit them to a stable, public repository and provide us with the relevant URLs, DOIs, or accession numbers. For a list of recommended repositories, please see https://journals.plos.org/plosone/s/recommended-repositories.

If there are ethical or legal restrictions on sharing a de-identified data set, please explain them in detail (e.g., data contain potentially sensitive information, data are owned by a third-party organization, etc.) and who has imposed them (e.g., an ethics committee). Please also provide contact information for a data access committee, ethics committee, or other institutional body to which data requests may be sent. If data are owned by a third party, please indicate how others may request data access.

RESPONSE: We have added an ethics statement to the manuscript mentioning the Mexican regulations and the approved protocol by our institution ethics committee (lines 139-142).

6. We note that Figure 1 in your submission contain [map/satellite] images which may be copyrighted. All PLOS content is published under the Creative Commons Attribution License (CC BY 4.0), which means that the manuscript, images, and Supporting Information files will be freely available online, and any third party is permitted to access, download, copy, distribute, and use these materials in any way, even commercially, with proper attribution. For these reasons, we cannot publish previously copyrighted maps or satellite images created using proprietary data, such as Google software (Google Maps, Street View, and Earth). For more information, see our copyright guidelines: http://journals.plos.org/plosone/s/licenses-and-copyright.

We require you to either (1) present written permission from the copyright holder to publish these figures specifically under the CC BY 4.0 license, or (2) remove the figures from your submission:

a. You may seek permission from the original copyright holder of Figure 1 to publish the content specifically under the CC BY 4.0 license.

We recommend that you contact the original copyright holder with the Content Permission Form (http://journals.plos.org/plosone/s/file?id=7c09/content-permission-form.pdf) and the following text:
“I request permission for the open-access journal PLOS ONE to publish XXX under the Creative Commons Attribution License (CCAL) CC BY 4.0 (http://creativecommons.org/licenses/by/4.0/). Please be aware that this license allows unrestricted use and distribution, even commercially, by third parties. Please reply and provide explicit written permission to publish XXX under a CC BY license and complete the attached form.”

Please upload the completed Content Permission Form or other proof of granted permissions as an ""Other"" file with your submission.

In the figure caption of the copyrighted figure, please include the following text: “Reprinted from [ref] under a CC BY license, with permission from [name of publisher], original copyright [original copyright year].”

b. If you are unable to obtain permission from the original copyright holder to publish these figures under the CC BY 4.0 license or if the copyright holder’s requirements are incompatible with the CC BY 4.0 license, please either i) remove the figure or ii) supply a replacement figure that complies with the CC BY 4.0 license. Please check copyright information on all replacement figures and update the figure caption with source information. If applicable, please specify in the figure caption text when a figure is similar but not identical to the original image and is therefore for illustrative purposes only.
The following resources for replacing copyrighted map figures may be helpful:

USGS National Map Viewer (public domain): http://viewer.nationalmap.gov/viewer/
The Gateway to Astronaut Photography of Earth (public domain): http://eol.jsc.nasa.gov/sseop/clickmap/
Maps at the CIA (public domain): https://www.cia.gov/library/publications/the-world-factbook/index.html and https://www.cia.gov/library/publications/cia-maps-publications/index.html
NASA Earth Observatory (public domain): http://earthobservatory.nasa.gov/
Landsat: http://landsat.visibleearth.nasa.gov/
USGS EROS (Earth Resources Observatory and Science (EROS) Center) (public domain): http://eros.usgs.gov/#
Natural Earth (public domain): http://www.naturalearthdata.com/

RESPONSE: We followed your suggestion in a previous version of the manuscript and this is mentioned in the legend of figure 1 as follows “Terrain maps were obtained from USGS EROS (Earth Resources Observatory and Science (EROS) Center) (public domain: http://eros.usgs.gov/#).”

7. Please review your reference list to ensure that it is complete and correct. If you have cited papers that have been retracted, please include the rationale for doing so in the manuscript text, or remove these references and replace them with relevant current references. Any changes to the reference list should be mentioned in the rebuttal letter that accompanies your revised manuscript. If you need to cite a retracted article, indicate the article's retracted status in the References list and also include a citation and full reference for the retraction notice.

RESPONSE: We have reviewed the reference list and we consider it is complete and correct. Thanks.

Additional Editor Comments:

Title: I would suggest removing "collected in Merida, Yucatan, Mexico " as the mouse model could be used to isolate ticks from other areas

RESPONSE: We agree with the Editor and modified the title but also added the that we isolated it from soft ticks, we hope she agrees with this change.

Line 61: remove "This includes wild animal nests or dens"

RESPONSE: We have removed this sentence as required.

Lines 135 and 192: remove "Sequences were deposited in GenBank.", being reported below. The same goes for the "Annotation pipeline....(accession numbers CP149102-CP149123)"

RESPONSE: We have removed these sentences as required.

Lines 248-249: Rewrite this sentence "These results show that A. puertoricensis is present in Mexico likely parasitizing opossums in urban neighborhoods", since as it stands it seems like a comment and not a result.

RESPONSE: We have modified this sentence by only mentioning that the ticks were located in urban parks and neighborhoods (line 252).

Lines 329-330: Change to "such as the vector ecology tick misidentification, lack of diagnostic tests and due to the difficulty culturing the bacteria"

RESPONSE: We have removed these sentences as suggested.

Reviewers' comments:

Reviewer's Responses to Questions

Comments to the Author

1. Is the manuscript technically sound, and do the data support the conclusions?

The manuscript must describe a technically sound piece of scientific research with data that supports the conclusions. Experiments must have been conducted rigorously, with appropriate controls, replication, and sample sizes. The conclusions must be drawn appropriately based on the data presented.

Reviewer #1: Yes

2. Has the statistical analysis been performed appropriately and rigorously?

Reviewer #1: N/A

3. Have the authors made all data underlying the findings in their manuscript fully available?

The PLOS Data policy requires authors to make all data underlying the findings described in their manuscript fully available without restriction, with rare exception (please refer to the Data Availability Statement in the manuscript PDF file). The data should be provided as part of the manuscript or its supporting information, or deposited to a public repository. For example, in addition to summary statistics, the data points behind means, medians and variance measures should be available. If there are restrictions on publicly sharing data—e.g. participant privacy or use of data from a third party—those must be specified.

Reviewer #1: Yes

4. Is the manuscript presented in an intelligible fashion and written in standard English?

PLOS ONE does not copyedit accepted manuscripts, so the language in submitted articles must be clear, correct, and unambiguous. Any typographical or grammatical errors should be corrected at revision, so please note any specific errors here.

Reviewer #1: Yes

5. Review Comments to the Author

Please use the space provided to explain your answers to the questions above. You may also include additional comments for the author, including concerns about dual publication, research ethics, or publication ethics. (Please upload your review as an attachment if it exceeds 20,000 characters)

Reviewer #1: The present study investigates if *Ornithodoros puertoricensis* ticks might act as vectors for *Borrelia puertoricensis*, not being previously reported in Ciudad Caucel.

Line 122: Is there an explanation to analyse such a low number of ticks (n=3)? This number might be low to emphasize if a result is reliable and to further perform statistics in the future. Please explain and clarify.

RESPONSE: Thank you for this comment/question. Only three specimens were used to extract DNA because the rest were used in the mouse model for the isolation of *Borrelia* sp. Moreover, they were all collected from the same park and morphologically they are similar same. We are confident that the results obtained with these specimens are representative for the other adult ticks. We have added a statement about this in lines 123 to 124.

Line 151: Please briefly describe how the exsanguination with a heparinized syringe to quantify and isolate the bacteria was performed

RESPONSE: We have added this information in the results section (lines 155 to 157).

Line 155: Please briefly describe how the samples were analyzed to evaluate the antibody response against Borrelia

RESPONSE: The reference quoted in this section has all the protocols. Given that we didn't present results on this we decided not to include the detailed protocol and only refer it. We hope that the reviewer agrees with us.

Line 156: Please specify what is a "chemically induced immunosuppression mouse model", and how these animals were "prepared" for the experiments

Response: The immunosupression of the mice is mentioned in lines 164 to 168 in the same section.

Line 171: Please briefly justify why the choice of the temperature at 35ºC to cultivate the bacteria, once the ticks temperature may vary accordingly to the environment (being even higher/lower than 35ºC). Please specify if other temperatures were used to test the best culture conditions.

Response: *Borrelia* sp. growth has been described in related papers by our labs and others to ocurr at 34 or 35 oC. In our labs we decided to use 35oC. We have added more references to support this. No other conditions were tested as Dr. Lopez lab has this protocol well standarized and in our lab in Mexico we reproduced it.

Line 173: Please specify which was the expected concentration of bacteria considered “enough” for the subculturing and DNA isolation. Only saying that “when the bacteria were detected” may not be enough to the reader to repeat the procedures.

Response: We apologize for this. We have added information to clarify this in lines 180 to 181.

Line 274: I wonder whether if the extraction with the phenol-clorophorm might represent a good choice to perform genome sequencing. Where the information related to the quality of the analysis are showed? The authors are invited to clarify these points somewhere on the paper.

Response: In our laboratories experiences (Job Lopez’s and Antonio Ibarra’s labs) the DNA is of great quality to continue for sequencing with the two platforms (Nanopore and Illumina) mentioned in the manuscript. We have added this information in lines 290-291.

Line 331: Please specify which are the “Recent improvements in culture medium formulations” that have been made lately to clarify for the reader.

RESPONSE: We have decided to remove this statement as the possibility of culturing Borrelia is not that recent. We apologize for this.

Line 332: Which may be the main reasons for the absence of which the accessible animals for the spirochete isolation? The authors are invited to describe these informations on the text.

RESPONSE: We have a modified this section and added more text that complements the following sentence (lines 336 to 340) together with information that was already in the manuscript (lines 340-344).

Line 374: Please mention a reference that mention this information “There is increasing evidence that B. puertoricensis is more prevalent in Latin America”.

RESPONSE: We have included a review reference that mentions the distribution of *Borrelia puertoricensis* in this region (line 382).

6. PLOS authors have the option to publish the peer review history of their article (what does this mean?). If published, this will include your full peer review and any attached files. If you choose “no”, your identity will remain anonymous but your review may still be made public.
Do you want your identity to be public for this peer review? For information about this choice, including consent withdrawal, please see our Privacy Policy.
Reviewer #1: No
