# Supplementary material for: Use of a mouse model for the isolation of Borrelia puertoricensis from soft ticks
Source: PLoS One. 2025 Feb 18;20(2):e0318652. doi: 10.1371/journal.pone.0318652 (PMC11835249; doi:10.1371/journal.pone.0318652)
Supplement: Attachment — Submitted filename: Rebuttal_letter_v2.docx [file pone.0318652.s001.docx]

**Rebuttal letter**

**PONE-D-24-31359**

**Use of a mouse model for the isolation of *Borrelia puertoricensis* from ticks collected in Merida, Yucatan, Mexico**

**PLOS ONE**

Dear Dr. Zueckert,

Thank you for your message regarding our submission to PLOS ONE. Please find attached the reponses to the reviewers and also the revised version of the manuscript including the changes asked to meet the journal requirements.

Responses are in bold letters and where appropriate the line number is indicated.

I look forward to hearing from you.

Best wishes,

J. Antonio Ibarra

Corresponding author

------------------------------------------------------------------------------

**Editor and journal comments:**

Dear Dr. Ibarra,

Thank you for submitting your manuscript to PLOS ONE. After careful consideration, we feel that it has merit but does not fully meet PLOS ONE’s publication criteria as it currently stands. Therefore, we invite you to submit a revised version of the manuscript that addresses the points raised during the review process.

All four reviewers found the study to be scientifically sound and of multifaceted interest for the scientific community. As for required modifications, all four reviewers noted multiple gaps in the description of the methods that need to be fully addressed in a revision. As one of the reviewers noted, this aspect of the study provides a novel simplified protocol for future studies, which makes those details absolutely necessary. Also, several reviewers request that Fig. 2 be revised or split into two separate figures to improve readability of Panel C. Reviewer 4 also provides a detailed list of small syntax changes that will improve consistency and readability of the manuscript, which should be quite easy address.

As for recommended revisions, Reviewer 3 noted that the title should be revised to better capture the wider scope of the manuscript, and that the abstract should be restructured for the same reason. Since I tend to agree and recommend that you revise this section as well to further improve the quality and potential reach of the manuscript.

Please submit your revised manuscript by Oct 10 2024 11:59PM. If you will need more time than this to complete your revisions, please reply to this message or contact the journal office at plosone@plos.org. When you're ready to submit your revision, log on to https://www.editorialmanager.com/pone/ and select the 'Submissions Needing Revision' folder to locate your manuscript file.

Please include the following items when submitting your revised manuscript:

**A rebuttal letter** that responds to each point raised by the academic editor and reviewer(s). You should upload this letter as a separate file labeled **'Response to Reviewers'.**

**A marked-up copy** of your manuscript that highlights changes made to the original version. You should upload this as a separate file labeled 'Revised Manuscript with Track Changes'.

**An unmarked version** of your revised paper without tracked changes. You should upload this as a separate file labeled 'Manuscript'.

**If you would like to make changes to your financial disclosure, please include your updated statement in your cover letter**. Guidelines for resubmitting your figure files are available below the reviewer comments at the end of this letter.

If applicable, we recommend that you deposit your laboratory protocols in protocols.io to enhance the reproducibility of your results. Protocols.io assigns your protocol its own identifier (DOI) so that it can be cited independently in the future. For instructions see: https://journals.plos.org/plosone/s/submission-guidelines#loc-laboratory-protocols. Additionally, PLOS ONE offers an option for publishing peer-reviewed Lab Protocol articles, which describe protocols hosted on protocols.io. Read more information on sharing protocols at https://plos.org/protocols?utm_medium=editorial-email&utm_source=authorletters&utm_campaign=protocols.

We look forward to receiving your revised manuscript.

Kind regards,

Wolfram Zueckert, Ph.D.

Academic Editor

PLOS ONE

---------------------------------------&---------------------------------------

**Journal Requirements:**

When submitting your revision, we need you to address these additional requirements.

1. Please ensure that your manuscript meets PLOS ONE's style requirements, including those for file naming. The PLOS ONE style templates can be found at

https://journals.plos.org/plosone/s/file?id=wjVg/PLOSOne_formatting_sample_main_body.pdf and

https://journals.plos.org/plosone/s/file?id=ba62/PLOSOne_formatting_sample_title_authors_affiliations.pdf

**Response: We have complied with this request by following the suggested instructions.**

2. In your Methods section, please provide additional information regarding the permits you obtained for the work. Please ensure you have included the full name of the authority that approved the field site access and, if no permits were required, a brief statement explaining why.

**Response: We have added the requested permit for the tick collection (lines 102-104). A brief statement was added in lines 104-105.**

3. Please include a complete copy of PLOS’ questionnaire on inclusivity in global research in your revised manuscript. Our policy for research in this area aims to improve transparency in the reporting of research performed outside of researchers’ own country or community. The policy applies to researchers who have travelled to a different country to conduct research, research with Indigenous populations or their lands, and research on cultural artefacts. The questionnaire can also be requested at the journal’s discretion for any other submissions, even if these conditions are not met. Please find more information on the policy and a link to download a blank copy of the questionnaire here: https://journals.plos.org/plosone/s/best-practices-in-research-reporting. Please upload a completed version of your questionnaire as Supporting Information when you resubmit your manuscript.

**Response: We need to let you know that the study was carried out completely in Mexico and as such we did not travel to another country for sampling. Our collaborators at the Baylor College of Medicine only processed the DNA samples. In spite of the latter, we have completed the questionnaire and submitted together with the revised version of the manuscript.**

4. Thank you for stating the following financial disclosure:

"This work was supported by funds to JAI from Secretaría de Investigación y Posgrado-IPN (20230850, 20240122, 20241496) and by funds provided to JEL from the National School of Tropical Medicine at Baylor College of Medicine and by funds."

**Please state what role the funders took in the study. If the funders had no role, please state:** "The funders had no role in study design, data collection and analysis, decision to publish, or preparation of the manuscript."

If this statement is not correct you must amend it as needed.

Please include this amended Role of Funder statement in your cover letter; we will change the online submission form on your behalf.

**Response: Thank you for updating the online statement. The suggested sentence was not added to the manuscript as the funding was removed in the revised version (see below) and it will appear once the paper is published.**

5. Thank you for stating the following in the Acknowledgments Section of your manuscript:

"We deeply thank Dr. Lilia Dominguez-López and Dr. José Luis González-Quiroz for providing the mice for this study. This work was supported by funds to JAI from Secretaría de Investigación y Posgrado-IPN (20230850, 20240122, 20241496) and by funds provided to JEL from the National School of Tropical Medicine at Baylor College of Medicine and by funds."

**We note that you have provided funding information that is not currently declared in your Funding Statement. However, funding information should not appear in the Acknowledgments section or other areas of your manuscript**.

We will only publish funding information present in the Funding Statement section of the online submission form. **Please remove any funding-related text from the manuscript and let us know how you would like to update your Funding Statement**. Currently, your Funding Statement reads as follows:

"This work was supported by funds to JAI from Secretaría de Investigación y Posgrado-IPN (20230850, 20240122, 20241496) and by funds provided to JEL from the National School of Tropical Medicine at Baylor College of Medicine and by funds."

**Please include your amended statements within your cover letter; we will change the online submission form on your behalf.**

**Response: Thank you, we have removed the funding statement from the Acknowledgments section and we will include the statement in the cover letter.**

6. We note that your Data Availability Statement is currently as follows: [All relevant data are within the manuscript and its Supporting Information files.]

Please confirm at this time whether or not your submission contains all raw data required to replicate the results of your study. Authors must share the “minimal data set” for their submission. PLOS defines the minimal data set to consist of the data required to replicate all study findings reported in the article, as well as related metadata and methods (https://journals.plos.org/plosone/s/data-availability#loc-minimal-data-set-definition).

**For example, authors should submit the following data:**

- The values behind the means, standard deviations and other measures reported;

- The values used to build graphs;

- The points extracted from images for analysis.

Authors do not need to submit their entire data set if only a portion of the data was used in the reported study.

If your submission does not contain these data, please either upload them as Supporting Information files or deposit them to a stable, public repository and provide us with the relevant URLs, DOIs, or accession numbers. For a list of recommended repositories, please see https://journals.plos.org/plosone/s/recommended-repositories.

If there are ethical or legal restrictions on sharing a de-identified data set, please explain them in detail (e.g., data contain potentially sensitive information, data are owned by a third-party organization, etc.) and who has imposed them (e.g., an ethics committee). Please also provide contact information for a data access committee, ethics committee, or other institutional body to which data requests may be sent. If data are owned by a third party, please indicate how others may request data access.

**Response: As initially mentioned, “All relevant data are within the manuscript and its Supporting Information files” and we do not have any supporting material.**

**7. We note that [Figure 1] in** your submission contain [map/satellite] images which may be copyrighted. All PLOS content is published under the Creative Commons Attribution License (CC BY 4.0), which means that the manuscript, images, and Supporting Information files will be freely available online, and any third party is permitted to access, download, copy, distribute, and use these materials in any way, even commercially, with proper attribution. For these reasons, we cannot publish previously copyrighted maps or satellite images created using proprietary data, such as Google software (Google Maps, Street View, and Earth). For more information, see our copyright guidelines: http://journals.plos.org/plosone/s/licenses-and-copyright.

We require you to either (1) present written permission from the copyright holder to publish these figures specifically under the CC BY 4.0 license, or (2) remove the figures from your submission:

a. You may seek permission from the original copyright holder of Figure 1 to publish the content specifically under the CC BY 4.0 license.

We recommend that you contact the original copyright holder with the Content Permission Form (http://journals.plos.org/plosone/s/file?id=7c09/content-permission-form.pdf) and the following text:

“I request permission for the open-access journal PLOS ONE to publish XXX under the Creative Commons Attribution License (CCAL) CC BY 4.0 (http://creativecommons.org/licenses/by/4.0/). Please be aware that this license allows unrestricted use and distribution, even commercially, by third parties. Please reply and provide explicit written permission to publish XXX under a CC BY license and complete the attached form.”

Please upload the completed Content Permission Form or other proof of granted permissions as an "Other" file with your submission.

In the figure caption of the copyrighted figure, please include the following text: “Reprinted from [ref] under a CC BY license, with permission from [name of publisher], original copyright [original copyright year].”

b. If you are unable to obtain permission from the original copyright holder to publish these figures under the CC BY 4.0 license or if the copyright holder’s requirements are incompatible with the CC BY 4.0 license, please either i) remove the figure or ii) supply a replacement figure that complies with the CC BY 4.0 license. Please check copyright information on all replacement figures and update the figure caption with source information. If applicable, please specify in the figure caption text when a figure is similar but not identical to the original image and is therefore for illustrative purposes only.

The following resources for replacing copyrighted map figures may be helpful:

USGS National Map Viewer (public domain): **http://viewer.nationalmap.gov/viewer/**

The Gateway to Astronaut Photography of Earth (public domain): **http://eol.jsc.nasa.gov/sseop/clickmap/**

Maps at the CIA (public domain): **https://www.cia.gov/library/publications/the-world-factbook/index.html** and **https://www.cia.gov/library/publications/cia-maps-publications/index.html**

NASA Earth Observatory (public domain): http://earthobservatory.nasa.gov/

Landsat: http://landsat.visibleearth.nasa.gov/

USGS EROS (Earth Resources Observatory and Science (EROS) Center) (public domain): http://eros.usgs.gov/#

Natural Earth (public domain): http://www.naturalearthdata.com/

**Response: We have opted for option “b” and used the USGS EROS website (lines 114-115). Thank you. We take this opportunity to let you know that these websites are not available at the links you provided: USGS National Map Viewer, The Gateway to Astronaut Photography of Earth, and Maps at the CIA, thus we suggest that you update them.**

8. Please review your reference list to ensure that it is complete and correct. If you have cited papers that have been retracted, please include the rationale for doing so in the manuscript text, or remove these references and replace them with relevant current references. Any changes to the reference list should be mentioned in the rebuttal letter that accompanies your revised manuscript. If you need to cite a retracted article, indicate the article’s retracted status in the References list and also include a citation and full reference for the retraction notice.

**Response: We have updated the reference list after reviewers suggested to add a few extra.**

-----------------------------------------------------------------------

**Reviewer's Responses to Questions**

**Comments to the Author**

1. Is the manuscript technically sound, and do the data support the conclusions?

The manuscript must describe a technically sound piece of scientific research with data that supports the conclusions. Experiments must have been conducted rigorously, with appropriate controls, replication, and sample sizes. The conclusions must be drawn appropriately based on the data presented.

Reviewer #1: Yes

Reviewer #2: Yes

Reviewer #3: Yes

Reviewer #4: Yes

2. Has the statistical analysis been performed appropriately and rigorously?

Reviewer #1: N/A

Reviewer #2: Yes

Reviewer #3: N/A

Reviewer #4: N/A

3. Have the authors made all data underlying the findings in their manuscript fully available?

The PLOS Data policy requires authors to make all data underlying the findings described in their manuscript fully available without restriction, with rare exception (please refer to the Data Availability Statement in the manuscript PDF file). The data should be provided as part of the manuscript or its supporting information, or deposited to a public repository. For example, in addition to summary statistics, the data points behind means, medians and variance measures should be available. If there are restrictions on publicly sharing data—e.g. participant privacy or use of data from a third party—those must be specified.

Reviewer #1: Yes

Reviewer #2: Yes

Reviewer #3: Yes

Reviewer #4: Yes

4. Is the manuscript presented in an intelligible fashion and written in standard English?

PLOS ONE does not copyedit accepted manuscripts, so the language in submitted articles must be clear, correct, and unambiguous. Any typographical or grammatical errors should be corrected at revision, so please note any specific errors here.

Reviewer #1: Yes

Reviewer #2: Yes

Reviewer #3: Yes

Reviewer #4: Yes

5. Review Comments to the Author

Please use the space provided to explain your answers to the questions above. You may also include additional comments for the author, including concerns about dual publication, research ethics, or publication ethics. (Please upload your review as an attachment if it exceeds 20,000 characters)

-----------------------------------------------------------------------

**Reviewers' comments:**

**Reviewer #1:** The manuscript describing the isolation of *Borrelia puertocensis* and its genomic characterization is an important finding on looking at the possible pathogens that can be transmitted by soft ticks. The manuscript is clearly written and all the figures are well presented. I have a few minor points that I would like the authors to address:

1. Lines127-128, the authors do not mention how many ticks were placed for feeding on each mouse and whether it was a mixture of adults and nymphs or separate.

**Response: We apologize for this, the number of ticks per mouse has been added to a modified version of table 1 (lines 143 and 218).**

2. Lines 147 and 148 and Lines 161-164, the authors describe inoculating media with the sera of infected mice. I am actually not aware of any work that has isolated spirochetes from sera. The work by Replogle et al., which the authors cite for this never used serum for inoculating/culturing of spirochetes. If this work has been done before , please cite the appropriate paper.

**Response: Reviewer is right, in the paper by Replogle et al. the authors use the media for culturing *Borrelia miyamotoi* and other Borreliae but not using serum. The use of serum from infected mice was described by our group in Bermudez et al. 2021 (doi: 10.1371/journal.pntd.0009642). This was added in line 170 and it was corrected in line 171.**

3. Line 26 , replace 'by' with 'in'.

**Response: Thank you for the correction, this has been done.**

4. Line 71, Replace 'Venzal's work' with 'Work by Venzal et al., ...."

**Response: The suggested change has been done.**

5. Line 127 - Correct 'groups; to 'grouped'.

**Response: The suggested change has been done (line 140).**

6. Line 136 - Remove 'a'

**Response: Thank you for the correction, this has been done (line 151).**

7. Line 155 - Missing company name for the HRP-conjugate recombinant protein G.

**Response: After one of the reviewer’s suggestion we have removed this section.**

8. Line 301 - Add 'in surveillance...."

**Response: The suggested change has been done (line 308).**

-----------------------------------------------------------------------

**Reviewer #2:** This is a technically sounds study demonstrating the isolation of a relapsing fever spirochete Borrelia puertoicensis, from argasid ticks in the Yucatan. Understanding the distribution of the disease agent and its vector is critical for educating public and physicians. With a couple of small modifications, this is a worthy addition to the literature.

Major comments:

Serology is described in methods, yet is not mentioned elsewhere in the paper.

**Response: We thank the reviewer for this observation and apologize for the mistake. This section has been removed from the revised manuscript as we did not intend to show these results.**

Please check sequence of 16s-1 primer- a quick blast search does not show any tick species

**Response: Oligonucleotides sequences were obtained from previous studies done by us and others based on those described by Black and Piesman (1994). We have double checked the specified sequence and indeed it is the one reported by them and used by multiple groups. After performing also a BLAST-n search it seems that oligo 16s-1 has identity with arthopods in general, not only with ticks, though it also has identity to, for instance *Amblyomma*. We hope this clarifies this issue.**

*Minor comment:*

The beginning of the introduction is repetitive (see lines 48 and 52)

**Response: We apologize for this, these lines have been reorganized (lines 56-61).**

-----------------------------------------------------------------------

**Reviewer #3:** (1) The title does not do justice to the comprehensiveness of the study. It was much more than just "isolation".

**Response: We thank the reviewer for her/his comments. We have changed the title for “Use of a mouse model for the isolation of *Borrelia puertoricensis* from ticks collected in Merida, Yucatan, Mexico” to better reflect the described work.**

(2) The Abstract is written in an informal style and could be more tightly structured and more fully informative about what was done.

**Response: We thank the reviewer for her/his suggestion. The abstract has been modified in the reviewed version of the manuscript.**

(3) More information on the dexamethasone injections, such as volume and diluent, would be helpful.

**Response: We apologize for not included this. We have added the information in line 158-162.**

(4) Line 161: Was the blood anti-coagulated? If so, how?

**Response: We apologize for not included this datum. We have added the information in line 150 and 167.**

(5) Figure 2 could be split into two separate figures. The scales of A/B and C are very different. It is difficult to read panel C without enlarging it, while that is not necessary for panels A and B. Alternatively: Do the authors really need that many taxa for panel C? No more than half that number should be sufficient. Reducing the number of taxa by half or more could allow all three panels to be in same figure.

**Response: We appreciate reviewer’s suggestion. Figure 2 has been change to include only ticks’ pictures and Figure 3 now shows the phylogenetic tree. We decided to do it this way because we believe that the field of soft ticks taxonomy is in need of more analyses like the one shown.**

(6) The authors should be expand the discussion of using dexamethasone for immunosuppression of the mice for propagating relapsing fever Borrelia species. Do they think that the appearance of neutralizing antibody is delayed or there is some effect on innate immunity? This is an important finding, since the use of MyD88 knockout mice or SCID mice is more expensive than using outbred mice treated with dexamethasone injections. This would also be more easily carried out where genetically immunodeficient mice are not ready at hand. They could also optionally briefly review other methods of acquired immunosuppression, including use of cytoxan mice and splenectomy, that investigators have used for propagating relapsing fever Borrelia to higher numbers in experimental animals.

**Response: We thank the reviewer for their comments and we have added discussion including previous findings and also discussing the putative role of dexamethasone in the used model (lines 367-379).**

-----------------------------------------------------------------------

**Reviewer #4:** The manuscript titled “Isolation of Borrelia puertoricensis from ticks collected in Merida, Yucatan, Mexico” describes the first isolation of this TBRF spirochete in Mexico and the first documentation of Alectorobius puertoricensis in the Yucatan Peninsula. RF isolates are important for providing clues of the geographic distribution and overall ecology of TBRF and the associated tick vectors, however, isolation can be challenging due to differences in host/lab model susceptibility and requirement of specialized culture media. Here, the authors utilized an easily implemented and inexpensive method to chemically immunosuppress lab mouse strains and were able to achieve high enough spirochetemia to achieve culture and isolation. This study presents several important findings: 1) an animal model that can be more easily sustained in areas with limited resources and funds to purchase or maintained specialized genetically immunosuppressed laboratory mouse strains, 2) new records of RF spirochete B. puertoricensis and associated tick vector A. puertoricensis, 3) identification of genetically unique BipA protein that is could be used for species specific serosurvey, and 4) documented host association (opossum) that will inform potential distribution and possibly peridomestic public health risk. Taken together the data presented here will undoubtedly improve the ability to conduct impactful TBRF research in Latin American and to ultimately better understand and decrease human disease risk. The manuscript is straightforward and well written.

**Response: We thank the reviewer for his/her comments remarking the relevance of our work.**

Specific comments:

In general, it would be helpful if the description of the study site could remain consistent throughout the paper. It is referred to as Merida, Ciudad Caucel, etc. After the initial description, could simply state, hereafter referred to as Ciudad Caucel. Lines 29, 42, 78, 88, Fig 1, 205-207, 282

**Response: We have made the suggested changes in the manuscript.**

Line 29: change to “…was isolated using an…”

**Response: As per another reviewer’s request the abstract has been modified.**

Line 37-38: move word genus to in front of *Borrelia*

**Response: The abstract has been changed we hope this one is better than the prwvious one.**

Line 70: please include a reference after “…has been previously misidentified as O. talaje”

**Response: The reference regarding this has been included (line 81).**

Fig 1: could you shade Yucatan black, as it is difficult to distinguish on the inset map

**Response: Yucatan is marked already in black in the original figure but we reckon it was very small. Therefore it was anlarged a little bit. We hope this version is better.**

Line 119-120, 184: can you explain the “highest value” and how it differs from the e-value, which was identical.

**Response: This was a mistake from our part, we apologize for this and thank the reviewer for noting this mistake.**

Line 127: change to “Ticks were grouped in pools…”

**Response: We have corrected the typo, thank you (line 140).**

Line 127-141: this section should be edited to include the details provided in the results and discussion regarding which mice were used in each of the two separate experiments. As written, it is not clear which mice were given Dex. It would be helpful to see a table of which mice strains were fed on by how many ticks, and the spirochetemias developed, numbers of infected but non-culturable animals etc. Line 129 states 27 animals were used, and in the results, low spirochetemias were observed in 4 mice and then four more mice were used for mechanical immunosuppression, so no spirochetemia observed in 19 mice?

**Response: We apologize for this confussion. We describe both protocols, one with non-treated mice and one treated with DEX. We hoped that the results section would be clear but it seems that we failed, so we have edited the methods section a little bit to clarify this. We hope that the corrected version is better (lines 137 to 164).**

Line 136: remove “a” after administering

**Response: We have corrected the typo, thank you.**

Line 138, 147: did euthanasia precede exsanguination? Please add /mention euthanasia

**Response: Yes, euthanasia preceded exsanguination and it is as described in this section.**

Line 148: please add details on number of mice and strains of mice used for chemical immunosuppression

**Response:**

**We have added the requested information in line142 and table 1.**

Line 173: small “p” in polymerase

**Response: We have corrected the typo, thank you (line 179).**

Line 208: remove scientific name

**Response: We consider this is an important datum to have as at least another opossum species lives in this state.**

Line 218; Table 1: Please add additional details to table caption including species and study location/year (captions should stand alone)

**Response: As mentioned in the Methods section, all the specimens were collected the same day on January 2023. We have added the number of den and captions mentioning this, related to Fig 1 for the den number and also the species identified. We hope this is OK.**

Fig 2: Panel C is unreadable, and the gray box is not visible

**Response: We apologize for this, as suggested by another reviewer we have added a new figure with a clearer version of the tree. We hope now it looks better.**

Line 234: how many bootstrap replicates?

**Response: We have added the requested information in the legend, it was 1000 bootstraps (line 251).**

Line 235: add “from this study” or the equivalent to the end of the sentence “…letters are the sequences of the analyzed ticks (from this study).”

**Response: We have added the suggested word in the actual Fig 3 legend in the revised manuscript (line 252).**

Line 236-237: please add the number of substitutions represented by the scale bar

**Response: We have alarged the image in a new figure and also added this information in the legend (line 248-254).**

Line 246: add Veracruz in ( ) after Mexico

**Response: We have added the suggested change (line 332).**

Line 251: what strains of mice did you observe low spirochetemias in?

**Response: We have added this information in a new version of table 1.**

Line 304; Table 2: Please add additional details to table caption including species and study location/year (captions should stand alone)

**Response: We have modified this table to include the requested information plus more information requested by the other reviewers.**

Line 322: add “isolating” before RF spirochetes

**Response: We have chagned the sentence (line 339).**

Line 323: change to continues to be a challenge

**Response: We have corrected the typo, thank you (line 340).**

Line 324: change to “where genetically immunosuppressed mice strains are complicated to maintain, such as in many Latin American and other resource-limited countries.”

**Response: We have added the suggested changes (lines 341-343).**

Line 359: incomplete, the sentence ends without a description of the funds.

**Response: As per journal policy, this section has been modified.**

Line 385: not sure if the 2947 is a typo, if not, missing date.

**Response: Thank you for this observation, it has been corrected (line 428).**

Line 388: lower case F in From "Southern America"

**Response: We have corrected the typo, thank you (line 431).**

6. PLOS authors have the option to publish the peer review history of their article (what does this mean?). If published, this will include your full peer review and any attached files.

If you choose “no”, your identity will remain anonymous but your review may still be made public.

**NO**

Do you want your identity to be public for this peer review? For information about this choice, including consent withdrawal, please see our Privacy Policy.

Reviewer #1: No

Reviewer #2: No

Reviewer #3: Yes: Alan G. Barbour

Reviewer #4: No
